# Voluminous bilateral adnexal cysts in a young female: the challenge of fertility preservation

**DOI:** 10.5935/1518-0557.20220061

**Published:** 2023

**Authors:** Diana Rodrigues-Martins, Fabiana Castro, Fernanda Costa, Diana Melo Castro

**Affiliations:** 1 Department of Women´s Health and Reproductive Medicine, Centro Materno-Infantil do Norte, Centro Hospitalar Universitário do Porto, Largo da Maternidade de Júlio Dinis, Porto, Portugal; 2 Departmet of Gynaecology and Obstetrics, Centro Hospitalar do Tâmega e Sousa, Penafiel, Portugal

**Keywords:** fertility preservation, adnexal cysts, teratoma, cystadenoma, ovarian insufficiency

## Abstract

An ovarian benign cyst is a common finding in women of reproductive age. However both the disease and its treatment may have an impact on ovarian reserve, resulting in a significant risk of premature ovarian insufficiency. The counselling on fertility preservation is of paramount importance in such cases. We report the management of a young woman with giant bilateral benign adnexal cysts, highlighting the complexity of fertility preservation in such scenario.

## INTRODUCTION

An ovarian cyst is a common finding in women of reproductive age (Legendre *et al*., 2014). Although mostly of benign origin, complications may arise, and the surgical removal may be life threatening (Leite *et al*., 2020). Additionally, significant long-term health implications of the disease and its treatment on female fertility, as well as psychological and hormonal health have been demonstrated (Yasui *et al*, 2012).

The diagnosis of adnexal tumours during the second and third decades of women’s life enhances the complexity of management, since the majority of the patient’s hadn´t already completed their reproductive plan (Mateo-Sánez *et al*., 2020). In such situations, fertility preservation (FP) should turn out as a priority within the treatment plan (Mateo-Sánez *et al*., 2020). Current guidelines recommend an ovary-sparing surgery (OSS) as the preferred surgical intervention for the treatment of benign ovarian cysts in women of fertile age (RCOG, 2012). The published literature underlines that the future reproduction and fertility related information should ideally be addressed early in the management, to ensure that the patient and the gynaecologist have the opportunity to discuss fertility aspects related to the surgery (Lind *et al*., 2013).

However, when facing a huge mass saving the ovarian tissue may be difficult (Mateo-Sánez *et al*., 2020). Finding the right balance between optimal tumour resection and maximal fertility preservation is especially challenging when it comes to a bilateral presentation (Braungart *et al*., 2020).

In this paper we report the management of giant bilateral benign adnexal cysts in a young woman, highlighting the complexity of fertility preservation in such scenario.

## CASE DESCRIPTION

A 23-year old female was referred to our centre due to voluminous intra-abdominal cysts of adnexal origin. The diagnosis was made by pelvic ultrasound performed because of a progressive abdominal distention. Her medical history was not remarkable. As for gynaecological history, she was nulliparous, the menarche was at age 11, and she had regular menstrual cycles. Her contraception method was condom.

**Figure 1:**
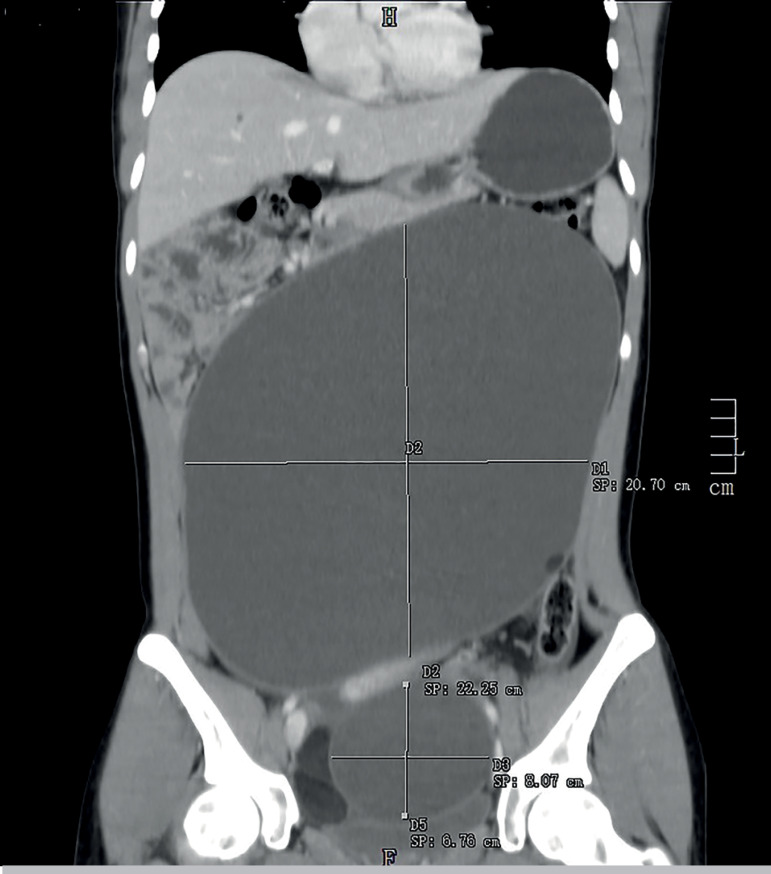
Coronal view of the computed tomography scan showing the large size of the cysts and its effects on the surrounding structures.

The bimanual examination revealed a distended abdomen, and a palpable cystic mass at the posterior vaginal fornix. A computed tomography scan showed a cystic lesion occupying the majority of the abdominal cavity, sized 20x11x27 centimetres (cm), compatible with a dermoid cyst of the left ovary. Another cystic lesion suggestive of a dermoid cyst of about 6x8 cm was present at the right ovary (Figure 1). The patient’s tumour markers including alpha fetoprotein, beta-human chorionic gonadotropin, carcinoembryonic antigen, cancer antigen 125, cancer antigen 15-3 and cancer antigen 19-9 were all within the normal ranges.

After discussion with the patient about the treatment options and possible implications over fertility, she was referred to fertility specialists. The possibility of oocyte cryopreservation prior to surgical treatment was discussed, however it ended up no being an option, due the risks involved, and the low likelihood of success.

**Figure 2:**
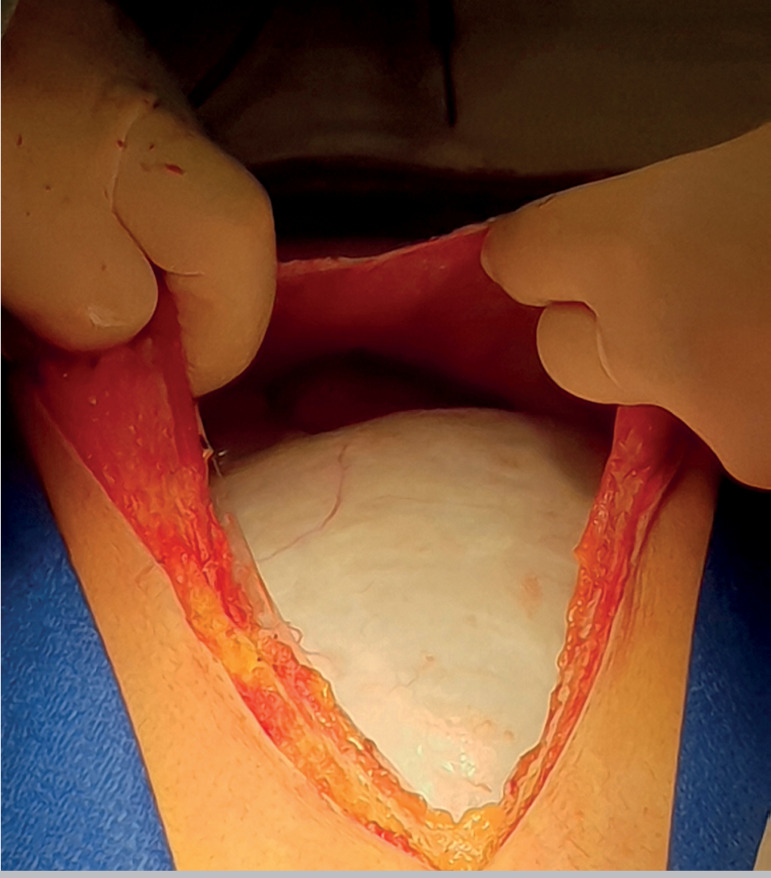
Photograph taken when entering the abdominal cavity. The cyst from the left adnexa occupied all the peritoneal cavity (more than 20 centimeters wide).

**Figure 3:**
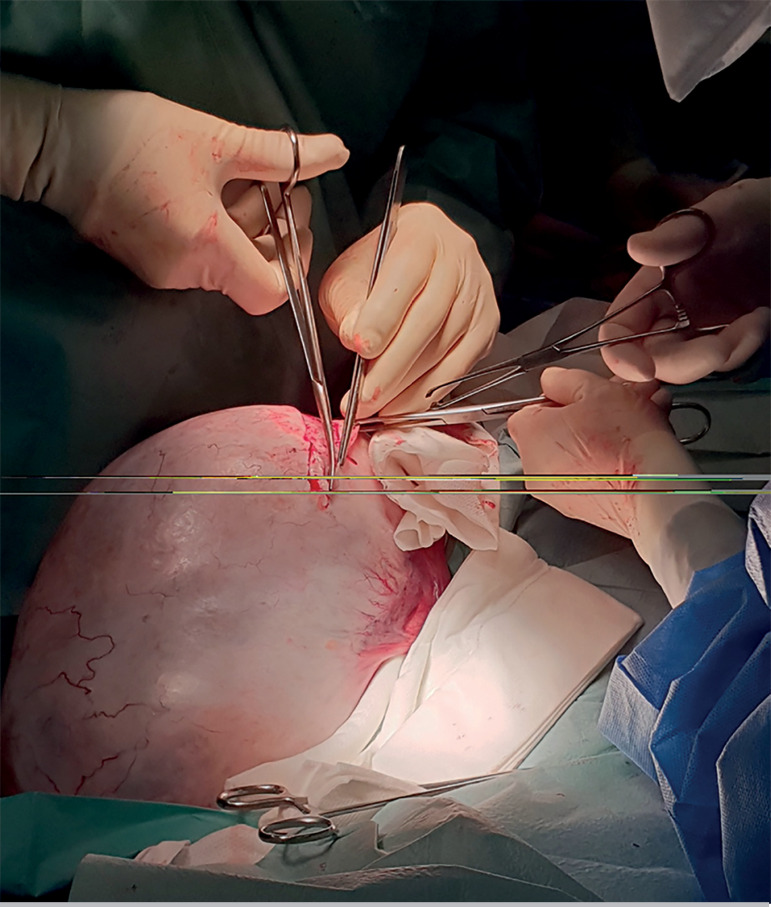
Enucleation of the cyst by the presence of a dissection plane between the tumour margins and the ovarian tissue.

**Figure 4:**
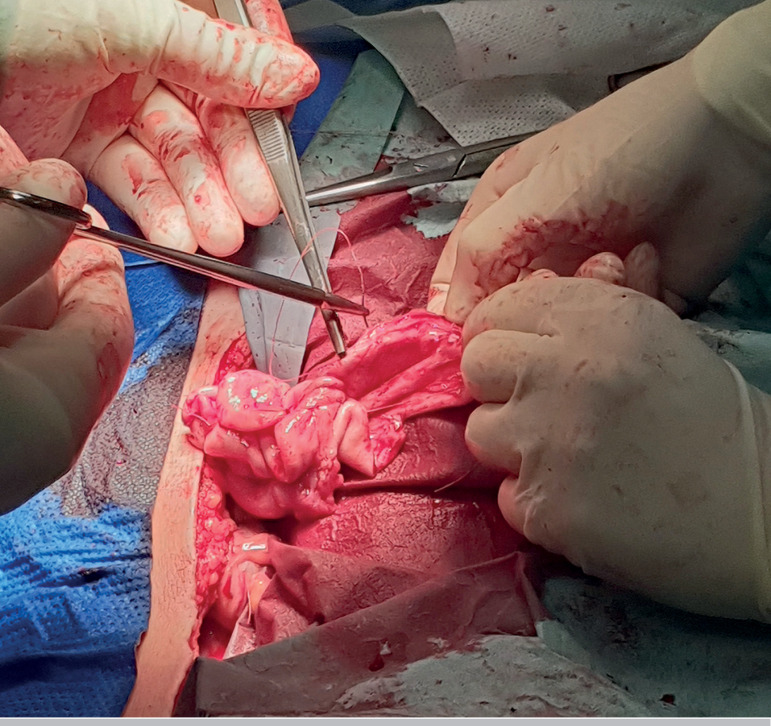
After the cystectomy was performed, the remaining ovarian tissue was very thin. Haemostasis was carefully done, and electrosurgical coagulation was avoided.

**Figure 5:**
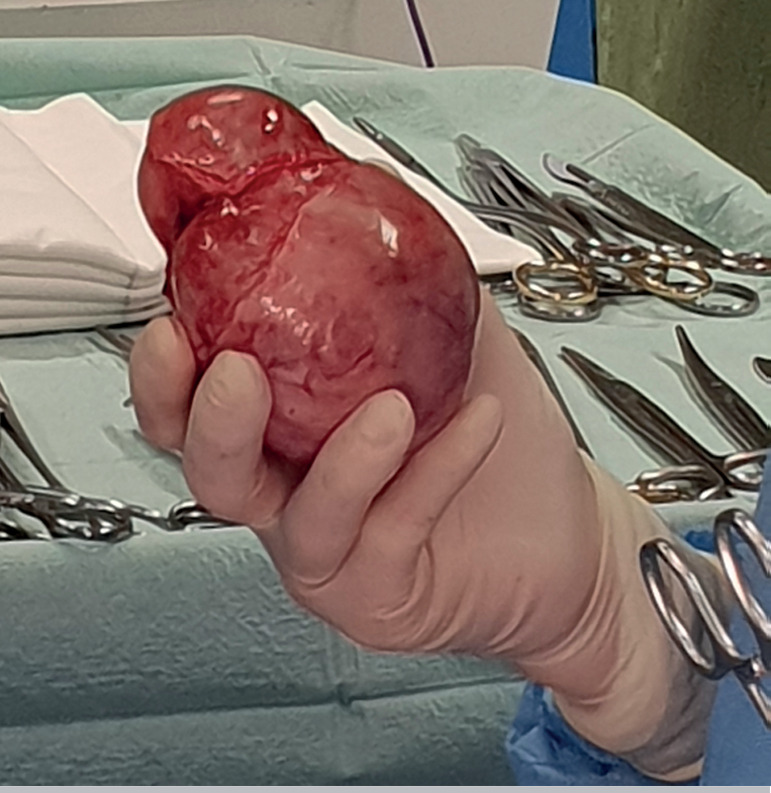
The larger cyst arising from the right ovary after complete enucleation.

The patient underwent an exploratory laparotomy with an intended conservative approach. Soon after a midline incision was performed, a huge cystic mass could be seen (Figure 2). Due to the existence of a dissection plane between the tumour margins and the ovarian tissue, the enucleation of the lesion arising from the left ovary was performed, and electrosurgical coagulation was avoided (Figures 3 and 4). Despite all the efforts, the spillage of the cyst contents was not prevented. Several smaller cysts were enucleated from the right ovary (Figure 5). The extemporaneous examination of all lesions was suggestive of a benign origin.

The histological analysis confirmed the presence of bilateral dermoid cysts. The lesion from the left ovary was also associated with a seromucinous cystadenoma. Ovarian tissue was not seen during the evaluation.

**Figure 6:**
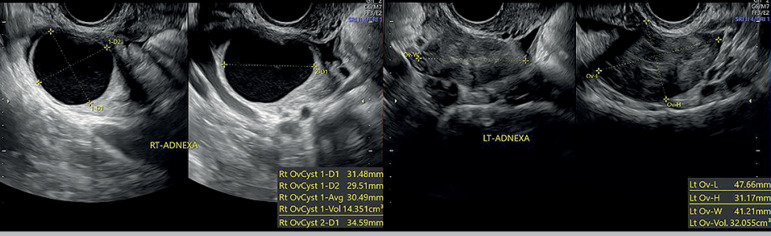
Pelvic ultrasound performed during the follicular phase three months after surgery. Despite of the ovary volumes achieved, only two and one antral follicles were displayed, on the left and right ovaries respectively. On the right ovary a cystic lesion could be seen. RT- right; LT- left.

The patient was discharged from the hospital without any complication on the second day after surgery. Two months later, she was asymptomatic and with regular menses. The patient´s serum anti-Müllerian hormone was evaluated 43 days after the surgery, with a measured value of 0.16 IU/mL (reference range of 1.0-9.71). A pelvic ultrasound was performed during the follicular phase of the third postoperative month, which showed a low follicular count on both ovaries, and a cystic lesion of about three cm in the right ovary (Figure 6). The patient was prompt referenced to fertility preservation specialists.

## DISCUSSION

Ovarian tumours in women of childbearing age are commonly seen, particularly the mature teratoma or dermoid cyst as known as well (Mateo-Sánez *et al*., 2020; Abdel-Hady *et al*., 2012).When an apparently benign ovarian cyst or tumour requires surgical excision, a conservative approach as ovarian cystectomy is advocated (Baksu *et al*., 2006), since it has been shown that unilateral oophorectomy increases the risk of premature ovarian failure and early menopause (Yasui *et al*., 2012). However, when a huge cyst is encountered preserving the ovarian tissue may be difficult (El-Agwany, 2018). It is particularly challenging when it comes to a bilateral presentation (Mateo-Sánez *et al*., 2020).

In this case of a young women with bilateral huge ovarian cysts, efforts were made to perform an OSS. The enucleation of all the macroscopic lesions were carried out, and preservation of ovarian tissue was achieved. As previously reported, in patients with a tumour size larger than 15 cm, the normal ovary becomes very thin (Oue *et al*., 2015), and the volume of ovarian tissue is substantially reduced after surgery (Baksu *et al*., 2006). However Reddy and Laufer demonstrated that after cystectomy the affected ovary resumed its normal size and volume despite the attenuated appearance of the ovarian cortex at the time of surgery (Reddy & Laufer, 2009). This is supported by the findings of this patient´s ultrasound performed after three months. The existence of a dissection plane between the tumour margins and the ovarian tissue, as in this case, seems to be the main factor associated with ovarian preservation (Oue *et al*., 2015).

Cyst’s enucleation techniques aim to be fertility-sparing, however, there is a discussion on the consequences of conservative surgery over endogenous hormone production, and fertility (Lind *et al*., 2015). The size and nature of the cyst being removed, bilaterally and/or repeated surgery, method of haemostasis and the skill and experience of the surgeon are all important factors that will determine how much of an effect, if any, the cystectomy will have on the ovarian reserve (Balachandren *et al*., 2021). It is important to highlight that the lesion inflicted to the ovarian stroma and vascularization by electrosurgical coagulation during haemostasis may have a substantial impact (Li *et al*., 2009). As performed in this case, bipolar energy should be preferred to monopolar, and coagulation should be as parsimonious and as selective as possible (Li *et al*., 2009).

It is not, however, easy to determine the effect of the cyst or a cystectomy on a woman’s future fertility, since the accuracy of ovarian reserve assessments, in the presence of ovarian cysts has not been well studied (Balachandren *et al.,* 2021). According to the results published by Lind *et al*. serum anti-Müllerian hormone (AMH) levels may be used as an indicator of ovarian reserve following ovarian cystectomy. The authors concluded that the reduction in AMH levels differed depending on the histopathological diagnosis, and that the cyst itself might have had a negative impact on ovarian physiology, as indicated by the increase in serum AMH levels postoperatively in some patients (Lind *et al*., 2015). Kim *et al*. (2013) compared the AMH levels in women with unilateral and bilateral dermoid cysts, with those of controls and found no significant difference. Since we did not performed ovarian reserve assessments before the surgery, we can only speculate on the impact of the disease and/or surgery on this regard.

When planning surgery of ovarian cysts, information about future reproduction and fertility plans should ideally be addressed early in the process (Lind *et al*., 2013). The risks of recurrence/metachronous disease and implications of oophorectomy should be talked about, in order to outline a personalized management strategy for each patient (Braungart *et al*., 2020). The importance of this subject should be underlined. In a study by Lind *et al*. more than two thirds of the patients reported a desire for having children in the future; however only half of women recalled receiving information about a possible impact of the surgery on fertility (Lind *et al*., 2013).

The present case was pre-operatively assessed by a mul-tidisciplinary team, including fertility experts. Oocyte retrieval for FP had not been possible before surgery; however it remained as part of the plan soon after the intervention. Although the patient maintained regular menses after surgery, the assessment of AMH levels, and the antral follicular count suggests that the ovarian reserve was compromised. We know that an incomplete surgical resection cannot be excluded, but the presence of an adnexal cyst on ultrasound soon after the procedure is a reminder of the possibility of recurrence in the remaining ovary. Young age (less than 30 years), large cyst size (greater than or equal to eight centimeters), and bilateral cysts were significant predictive factors for dermoid cysts recurrence (Fibus, 2000).

In conclusion, this case illustrates the challenge that the treatment of benign adnexal pathology in young women can represent, especially when taking into account the importance of FP. Given the risk of recurrence and premature ovarian failure, counselling and FP should be prioritized, in a prompt and quick manner.
